# Patient Selection for Partial Breast Irradiation in the Era of Sentinel Lymph Node Biopsy Omission: A Narrative Review

**DOI:** 10.3390/cancers18172745

**Published:** 2026-08-24

**Authors:** Efstratios Karagiannis, Roger von Moos, Alessia Lardi, Silke-Gerda Brockmann, Peter Martin Fehr, Tobias Freyholdt, Ursula Hasler-Strub, Daniel Krause, Martina Maranta, Riccardo Lorenzini, Mirija Wüster, Barbara-Christina Padberg Sgier, Sophie Reinhart, Alexander Rieke, Stefan Kneifel, Johann Schönhofer, Michael Schwitter, Robert Wenger, Marc Wermke, Winfried Arnold

**Affiliations:** 1 Department of Radiation Oncology, Kantonsspital Graubünden, 7000 Chur, Switzerland; silkegerda.brockmann@ksgr.ch (S.-G.B.); winfried.arnold@ksgr.ch (W.A.); 2 Department of Medical Oncology/Hematology, Kantonsspital Graubünden, 7000 Chur, Switzerland; roger.vonmoos@ksgr.ch (R.v.M.); ursula.hasler-strub@ksgr.ch (U.H.-S.); sophie.reinhart@ksgr.ch (S.R.); michael.schwitter@ksgr.ch (M.S.); 3 Department of Plastic Surgery, Kantonsspital Graubünden, 7000 Chur, Switzerland; alessia.lardi@ksgr.ch (A.L.); riccardo.lorenzini@ksgr.ch (R.L.); robert.wenger@ksgr.ch (R.W.); 4 Department of Obstetrics and Gynecology, Kantonsspital Graubünden, 7000 Chur, Switzerland; peter.fehr@ksgr.ch (P.M.F.); martina.maranta@ksgr.ch (M.M.); mirja.wuester@ksgr.ch (M.W.); 5 Department of Radiology, Kantonsspital Graubünden, 7000 Chur, Switzerland; tobias.freyholdt@ksgr.ch (T.F.); alexander.rieke@ksgr.ch (A.R.); johann.schoenhofer@ksgr.ch (J.S.); 6 Department of Pathology, Kantonsspital Graubünden, 7000 Chur, Switzerland; daniel.krause@ksgr.ch (D.K.); barbarachristina.padberg@ksgr.ch (B.-C.P.S.); 7 Department of Nuclear Medicine, Kantonsspital Graubünden, 7000 Chur, Switzerland; stefan.kneifel@ksgr.ch (S.K.); marc.wermke@ksgr.ch (M.W.)

**Keywords:** partial breast irradiation, sentinel lymph node biopsy omission, breast cancer, treatment de-escalation, axillary staging, occult nodal disease

## Abstract

Partial breast irradiation (PBI) and omission of sentinel lymph node biopsy (SLNB) are major advances in the de-escalation of locoregional treatment for early-stage breast cancer. These strategies have evolved independently under different clinical assumptions. The randomized trials establishing PBI incorporated pathological axillary staging into patient selection, although the permitted nodal burden varied among studies, whereas contemporary trials have demonstrated that sentinel lymph node biopsy (SLNB) can be safely omitted in carefully selected patients. Consequently, radiation oncologists are increasingly encountering otherwise suitable candidates for PBI who lack pathological nodal information because SLNB has been omitted, creating an important evidence gap. This narrative review examines how pathological nodal staging became embedded within PBI eligibility criteria and whether this long-standing requirement remains justified following the introduction of SLNB omission. We discuss the biological and clinical rationale underlying current patient selection for PBI, the limitations of extrapolating evidence from whole-breast irradiation to PBI, and the potential contribution of modern imaging, tumor biology, molecular profiling, and prediction models for occult nodal disease to individualized risk assessment. Current evidence does not justify routine implementation of PBI following omission of SLNB. Pathological nodal information may be considered as one component of integrated risk assessment rather than an isolated determinant of PBI eligibility. Patients otherwise meeting contemporary radiotherapy omission criteria represent the most appropriate population for prospective evaluation of this combined de-escalation strategy. This review defines the resulting evidence gap and proposes a conceptual framework for future clinical investigation.

## Simple Summary

Partial breast irradiation is a well-established treatment for carefully selected patients with early-stage breast cancer. Until recently, these patients routinely underwent sentinel lymph node biopsy to determine whether cancer had spread to the axillary lymph nodes. However, recent clinical trials have shown that this operation can be safely omitted in selected patients without compromising cancer outcomes. This has created a new clinical situation in which otherwise suitable candidates for partial breast irradiation may no longer have pathological information about their lymph nodes. Whether these patients should automatically be excluded from partial breast irradiation remains unknown because this combination has never been prospectively studied. This review explains how this evidence gap has emerged, examines whether pathological lymph node assessment should remain an absolute requirement for patient selection, and identifies the most appropriate population for future clinical studies aimed at further personalizing and safely de-escalating breast cancer treatment.

## 1. Introduction

The history of early breast cancer treatment is one of progressive therapeutic de-escalation. Over the past four decades, successive generations of clinical trials have demonstrated that carefully selected patients can achieve equivalent oncological outcomes with less intensive locoregional treatment. This evolution has reshaped both breast surgery and radiation oncology, replacing radical interventions with more individualized strategies that preserve oncological efficacy while minimizing treatment-related morbidity and maintaining quality of life.

Radical mastectomy has largely been replaced by breast-conserving surgery [1,2], axillary lymph node dissection has been superseded by sentinel lymph node biopsy (SLNB) in clinically node-negative disease [3], and conventional whole-breast irradiation (WBI) has evolved toward (ultra)-hypofractionation [4,5] and, for selected patients, partial breast irradiation (PBI) [6,7,8,9]. More recently, routine SLNB has been challenged in patients with favorable clinicopathological characteristics, reflecting the broader shift toward individualized treatment balancing oncological benefit against treatment-related harm [10,11].

Among these advances, PBI represents one of the most successful examples of radiation treatment de-escalation. Multiple randomized trials have shown comparable local control between PBI and whole-breast irradiation in appropriately selected patients. PBI reduces irradiated volume and treatment duration and, in several studies, is associated with reduced late toxicity and improved cosmetic outcomes [6,7,8,9]. PBI is now established as a standard treatment option in major international guidelines for appropriately selected patients with early-stage breast cancer [12,13].

The success of PBI has depended not only on advances in radiation delivery but also on careful patient selection. The pivotal randomized trials predominantly enrolled patients with favorable clinicopathological characteristics, while pathological axillary staging was incorporated into patient selection despite differences in the permitted nodal burden [6,7,8,9].

While most established selection criteria remain largely undisputed, the role of pathological axillary staging deserves renewed consideration. The pivotal PBI trials were conceived and conducted during a period in which SLNB had become the standard method of axillary staging; consequently, pathological nodal information was routinely available and incorporated into patient selection and subsequent guideline recommendations [12,13]. Whether this information independently contributes to successful patient selection for PBI or partly reflects the surgical standard under which these trials were conducted remains largely unexplored.

The publication of the SOUND and INSEMA trials has brought this issue into sharper focus [10,11]. Both studies demonstrated excellent oncological outcomes with reduced treatment-related morbidity in carefully selected patients undergoing omission of SLNB. In SOUND, adjuvant radiotherapy was delivered according to institutional practice, with approximately 80% of patients receiving whole-breast irradiation, whereas INSEMA explicitly prohibited PBI and mandated whole-breast irradiation for all patients following breast-conserving surgery. These trials have therefore created a new clinical scenario in which otherwise suitable candidates for PBI may lack pathological nodal information because SLNB has been omitted. As a result, radiation oncologists are increasingly faced with this situation in routine practice. Whether pathological nodal staging should therefore remain an indispensable prerequisite for PBI, or whether patient selection can instead rely on clinical, radiological, pathological, and biological risk assessment remains an unanswered question.

This review examines how these parallel developments intersect in contemporary breast cancer management. We review the evolution of PBI eligibility criteria, the rationale underlying pathological nodal staging, and the implications of SLNB omission trials for patient selection. Finally, we discuss whether pathological nodal information should remain an indispensable prerequisite for PBI and outline potential directions for future risk-adapted patient selection.

## 2. Evolution of Patient Selection for Partial Breast Irradiation (PBI)

### 2.1. The Biological Basis of PBI

The concept of PBI originated from a growing understanding of the biological patterns of local recurrence following breast-conserving surgery. Pathological mapping studies indicated that the majority of ipsilateral breast tumor recurrences arise in close proximity to the original tumor bed, whereas lesions occurring elsewhere within the breast frequently represent biologically distinct new primary tumors rather than true local recurrences [14,15]. These observations challenged the long-standing assumption that irradiation of the entire breast was necessary for all patients undergoing breast-conserving therapy.

The possibility of reducing radiation volumes, however, depended on a fundamental prerequisite: identifying patients in whom residual microscopic disease was unlikely to extend beyond the immediate vicinity of the primary tumor. Patient selection became the cornerstone of the PBI concept, distinguishing it from whole-breast irradiation, which can be broadly applied following breast-conserving surgery.

Long before formal eligibility criteria were incorporated into randomized trials, clinicopathological features such as small tumor size, unifocal disease, negative surgical margins, favorable histology, and limited lymphovascular invasion had already emerged as markers of localized disease and low recurrence risk. These observations provided the biological foundation upon which contemporary PBI eligibility criteria were later built [14,15].

### 2.2. Clinical Validation and the Evolution of Patient Selection

The clinical development of PBI relied on a series of randomized trials designed not only to determine whether radiation treatment volumes could be safely reduced but also to identify the patients in whom such de-escalation could be achieved without compromising oncological outcomes. The development of PBI should be viewed as the progressive refinement of a low-risk clinical phenotype rather than simply the validation of a new radiation technique.

The restrictive eligibility criteria adopted in the earliest randomized PBI trials were not arbitrary but reflected the biological understanding of local recurrence. Investigators deliberately enrolled patients with the lowest probability of residual disease beyond the tumor bed, thereby creating a clinical model in which reduction in radiation volumes could be evaluated with acceptable oncological safety. As evidence accumulated, this initially pragmatic selection strategy progressively evolved into the eligibility criteria that continue to underpin existing guideline recommendations.

Confidence in PBI increased progressively as randomized trials consistently demonstrated that limiting irradiation to the tumor bed did not compromise oncological outcomes in carefully selected patients. This evidence emerged across different techniques and treatment schedules. The GEC-ESTRO trial established multicatheter brachytherapy as an effective alternative to whole-breast irradiation in a highly selected low-risk population [8]. Subsequently, IMPORT LOW showed that comparable oncological outcomes could be achieved using external-beam radiotherapy, substantially improving the accessibility and reproducibility of PBI [6]. The Florence trial further simplified treatment by demonstrating excellent long-term outcomes with an ultrahypofractionated five-fraction schedule [7], while NSABP B-39/RTOG 0413 explored broader eligibility criteria across multiple treatment techniques [9]. Despite important differences in radiation technique and study design, these trials consistently confirmed that excellent long-term local control could be achieved when PBI was restricted to appropriately selected patients [6,7,8,9]. The principal eligibility criteria, nodal staging requirements, treatment regimens, and long-term local outcomes of these pivotal PBI trials are summarized in Table 1.

Collectively, these studies accomplished more than validating different radiation techniques. Despite differences in treatment delivery, study design, and permitted nodal burden, they predominantly enrolled patients with favorable clinicopathological characteristics, while pathological axillary staging was incorporated into patient selection. These characteristics subsequently informed international guideline recommendations and continue to shape eligibility for PBI [12,13].

These characteristics should not be interpreted as independently validated predictors of PBI efficacy. Instead, they define the cumulative low-risk clinical phenotype repeatedly associated with excellent outcomes across successive randomized trials. Pathological nodal information occupies a unique position within this framework and warrants separate consideration. Understanding how it became embedded within PBI eligibility is essential before considering its role in the era of sentinel lymph node omission.

## 3. The Role of Pathological Nodal Staging in Patient Selection for PBI

Pathological axillary staging was incorporated into the eligibility assessment of the randomized trials that established PBI [6,7,8,9] and remains an important component of current international guideline recommendations [12,13]. Although the permitted nodal burden differed among trials, pathological nodal information was available for patient selection. Unlike tumor size, surgical margins, hormone receptor status, or other clinicopathological characteristics, pathological nodal status is not an intrinsic property of the primary tumor but the result of a surgical staging procedure. Consequently, its role in PBI eligibility criteria reflects biological considerations, the evolution of axillary management, and the radiotherapy strategies under which PBI was developed.

When the pivotal PBI trials were initiated, sentinel lymph node biopsy (SLNB) had already become the standard method of axillary staging for clinically node-negative breast cancer [3]. Pathological nodal information was therefore routinely available for patients undergoing breast-conserving surgery and naturally became incorporated into trial eligibility criteria. Under these circumstances, pathological axillary staging became embedded within PBI eligibility almost by default, rather than through prospective evaluation of its independent contribution to patient selection.

The radiotherapy context in which PBI was developed is equally important. The randomized trials compared PBI with whole-breast irradiation, the contemporary standard of care, which frequently delivered incidental irradiation to axillary levels I and II, potentially contributing to regional control in patients with occult nodal disease [16,17]. In contrast, PBI intentionally confines irradiation to the vicinity of the tumor bed and delivers little or no incidental treatment to the axilla [18]. Pathological nodal staging may assume greater importance when radiation is confined to the breast alone, as undetected nodal disease would not be expected to receive incidental irradiation. Accordingly, evidence supporting omission of SLNB in patients receiving whole-breast irradiation cannot automatically be extrapolated to patients treated with PBI.

These historical and radiobiological considerations explain why pathological nodal information became firmly embedded within PBI eligibility criteria. Whether this information remains indispensable in an era of modern imaging, biology-driven systemic therapy, and omission of SLNB is a different question entirely—and one that has not been addressed by existing randomized trials.

## 4. Evidence Supporting Sentinel Lymph Node Omission

### 4.1. Why Was SLNB Questioned?

Sentinel lymph node biopsy has long been regarded as the standard procedure for axillary staging in patients with clinically node-negative breast cancer [3]. Compared with axillary lymph node dissection, SLNB substantially reduced surgical morbidity while maintaining excellent staging accuracy and regional control. Nevertheless, the procedure remains associated with measurable complications, including lymphedema, pain, sensory disturbances, reduced shoulder mobility, and impaired quality of life [19]. As systemic therapy became largely guided by tumor biology rather than nodal burden, and improvements in breast imaging substantially reduced the likelihood of clinically occult axillary disease, the clinical value of routine pathological staging in all patients was increasingly questioned [20].

These developments gave rise to a new generation of randomized trials designed to determine whether axillary surgery could be safely omitted in carefully selected patients with biologically favorable, clinically node-negative breast cancer. Instead of asking whether SLNB accurately stages the axilla, these studies addressed a different question: Does the information obtained from routine pathological nodal staging meaningfully improve oncological outcomes in patients already expected to have a very low risk of regional recurrence [10,11]?

This distinction is fundamental. The omission trials were not designed to challenge the prognostic value of nodal status but rather to evaluate whether the benefits of routine pathological staging continue to outweigh its associated morbidity in an era of modern imaging, effective systemic therapy, and individualized local treatment.

### 4.2. SOUND

The SOUND trial represented the first randomized study specifically designed to evaluate omission of sentinel lymph node biopsy in patients with clinically and sonographically node-negative early breast cancer [10]. The underlying hypothesis was that modern preoperative axillary ultrasound, combined with favorable tumor biology and modern systemic therapy, could identify a patient population with such a low probability of clinically relevant nodal disease that routine pathological staging would no longer improve oncological outcomes.

Patient selection was intentionally restrictive. Eligible patients had small invasive breast cancers (cT1), negative axillary ultrasound, and were scheduled to undergo breast-conserving surgery. Adjuvant systemic therapy and radiotherapy were delivered according to routine institutional practice, reflecting routine clinical management rather than a protocol-driven radiation strategy.

After a median follow-up of 5.7 years, omission of SLNB was not associated with inferior distant disease-free survival, while significantly reducing surgical morbidity. These findings provided the first high-level evidence that routine pathological axillary staging may be safely omitted in carefully selected patients without compromising clinical outcomes.

From the perspective of PBI, several important considerations deserve emphasis. First, the study was not designed to evaluate radiation treatment de-escalation. Approximately 80% of patients received whole-breast irradiation, whereas only a small minority underwent PBI according to local institutional practice. Consequently, the trial provides robust evidence supporting surgical de-escalation but only limited information regarding the safety of combining SLNB omission with PBI. Second, the favorable outcomes observed in SOUND were achieved in a population that closely resembles the established low-risk phenotype traditionally considered suitable for PBI, highlighting the considerable overlap between patients selected for surgical and radiation treatment de-escalation.

### 4.3. INSEMA

The INSEMA trial substantially expanded the evidence supporting omission of sentinel lymph node biopsy by evaluating this strategy in a large population of patients with clinically node-negative early breast cancer undergoing breast-conserving surgery [11]. In contrast to SOUND, which primarily served as a proof-of-concept study, INSEMA was designed to determine whether omission of SLNB was non-inferior to SLNB in appropriately selected patients.

Eligibility criteria remained deliberately conservative and reflected a population with a low anticipated risk of axillary involvement. Patients underwent careful preoperative clinical and imaging assessment, while adjuvant systemic therapy was administered according to standard recommendations. The radiation protocol differed fundamentally from the clinical scenario addressed in the present review. Following breast-conserving surgery, whole-breast irradiation was mandated for all patients, whereas PBI was explicitly prohibited.

After a median follow-up of six years, omission of SLNB was associated with excellent oncological outcomes, demonstrating non-inferiority for invasive disease-free survival while significantly reducing surgical morbidity, including lymphedema, impaired arm mobility, and pain [11,21]. Together with the SOUND trial, these findings firmly established that routine pathological axillary staging can be safely omitted in carefully selected patients undergoing contemporary multidisciplinary treatment.

From the perspective of PBI, the principal limitation of INSEMA is straightforward: whole-breast irradiation was mandatory, whereas PBI was not permitted. Thus, the trial cannot determine whether omission of SLNB can be safely combined with PBI in otherwise low-risk patients.

Paradoxically, the greatest contribution of INSEMA may therefore lie not in answering this question, but in defining the next one. By demonstrating that omission of SLNB is safe in carefully selected patients while simultaneously excluding PBI, INSEMA defines the evidence gap at the intersection of surgical and radiation treatment de-escalation. Addressing this gap requires determining whether the biological characteristics that identify patients suitable for the omission of axillary surgery also identify those in whom PBI can be safely used without pathological nodal staging.

### 4.4. What Do SOUND and INSEMA Actually Tell Us About PBI?

Although SOUND and INSEMA have fundamentally changed the surgical management of clinically node-negative breast cancer, neither trial was designed to evaluate radiation treatment de-escalation [10,11]. Their findings cannot be directly extrapolated to patients receiving PBI. These studies provide several important observations that are highly relevant when reconsidering current PBI eligibility criteria.

First, both trials demonstrated that modern clinical and imaging assessment can identify a population in whom omission of pathological axillary staging is associated with very low rates of clinically relevant axillary recurrence. Patients enrolled in these studies share many of the favorable clinicopathological characteristics traditionally used to identify candidates for PBI, including small tumors, hormone receptor-positive disease, clinically node-negative axillae, and a low anticipated risk of locoregional recurrence. This substantial overlap suggests that surgical and radiation treatment de-escalation are increasingly targeting the same biological phenotype.

Second, both trials illustrate the changing role of pathological nodal staging in current breast cancer management. Historically, pathological confirmation of node-negative disease provided essential prognostic information and influenced recommendations for systemic therapy and regional irradiation [20]. However, as treatment decisions have become largely driven by tumor biology, genomic profiling, and high-quality imaging, the incremental clinical value of routine pathological staging has diminished for many patients with low-risk disease.

At the same time, an important limitation remains. Because omission of SLNB has been prospectively evaluated predominantly in patients receiving whole-breast irradiation, available evidence cannot determine whether pathological nodal staging becomes equally dispensable when radiation treatment is confined to the tumor bed. This distinction is fundamental when interpreting the absence of randomized data combining omission of SLNB with PBI.

Collectively, these findings challenge the assumption that pathological nodal information must remain an indispensable prerequisite for PBI patient selection. At the same time, they do not establish that pathological nodal staging can be safely omitted in all patients considered for PBI. Rather, they identify a previously unexplored clinical scenario in which otherwise suitable candidates for PBI undergo omission of SLNB. Whether pathological nodal information provides sufficient incremental value to justify excluding these patients from PBI remains unknown and cannot be answered by existing randomized trials.

## 5. Reconsidering Patient Selection for Partial Breast Irradiation (PBI) in the Era of Sentinel Node Omission

### 5.1. Should Contemporary PBI Eligibility Criteria Be Reconsidered?

The eligibility criteria currently used to select patients for PBI have proven remarkably robust over the past two decades. Derived from successive randomized trials [6,7,8,9] and incorporated into international guidelines [12,13], they have consistently identified a population with an exceptionally low risk of ipsilateral breast tumor recurrence. These criteria were established within a treatment paradigm in which pathological nodal information following sentinel lymph node biopsy was routinely available.

The introduction of SLNB omission challenges one of the historical assumptions underlying current PBI eligibility: that pathological nodal staging is available for every patient undergoing breast-conserving surgery. This does not question either the prognostic significance of nodal involvement or the evidence supporting current PBI eligibility criteria. Rather, it raises the question of whether pathological nodal information should continue to function as an absolute prerequisite for PBI when advances in breast imaging and biological risk stratification have fundamentally changed the management of early breast cancer.

Current de-escalation strategies have evolved along separate surgical and radiation pathways. Patients undergoing omission of SLNB continue to receive whole-breast irradiation, whereas patients receiving PBI have traditionally been required to undergo SLNB. Whether this separation continues to represent the optimal balance between oncological safety and treatment-related morbidity has never been prospectively evaluated and defines the central evidence gap addressed in this review.

The central issue is therefore not whether pathological nodal staging remains clinically relevant but whether its incremental contribution to patient selection is identical across all patients considered for PBI. It is plausible that, in carefully selected individuals with an otherwise exceptionally favorable clinical, imaging, pathological, and biological profile, pathological nodal staging provides relatively little additional information. This concept is also reflected in recent ASCO Clinical Insights, which acknowledge that PBI may be considered following SLNB omission in carefully selected patients with a very low probability of nodal involvement, emphasizing individualized multidisciplinary decision-making [22].

### 5.2. Patients Otherwise Meeting Contemporary Radiotherapy Omission Criteria: The Most Appropriate Candidates for Reconsideration of PBI Eligibility

The evolution of radiotherapy omission trials provides an important perspective on the future of patient selection for PBI. Studies such as CALGB 9343 and PRIME II first demonstrated that the omission of adjuvant radiotherapy is associated with only a modest increase in local recurrence among carefully selected older women with hormone receptor-positive, node-negative breast cancer receiving endocrine therapy [23,24]. More recently, prospective studies including LUMINA and IDEA have further refined this concept by incorporating biological and molecular characteristics, identifying patient populations with exceptionally low rates of local recurrence despite the omission of radiotherapy altogether [25,26]. Collectively, these studies illustrate the ongoing transition from anatomy-based toward biology-driven local treatment de-escalation. The key eligibility criteria, nodal assessment strategies, treatment characteristics, and outcomes of the SLNB omission and radiotherapy omission studies relevant to this proposed approach are compared in Table 2.

From the perspective of PBI, these studies define exceptionally low-risk populations based on increasingly refined clinical, pathological, and biological selection. Patients who otherwise meet these characteristics but undergo omission of SLNB represent the population in whom the incremental value of pathological nodal information for PBI selection deserves the greatest scrutiny.

An important qualification is required. Most of the radiotherapy omission studies discussed above, including PRIME II, LUMINA, and IDEA, included patients with pathologically confirmed node-negative disease, whereas CALGB 9343 required clinically node-negative disease and did not mandate axillary dissection. Patients undergoing omission of SLNB therefore cannot reproduce all of these trial populations exactly. The population proposed here instead refers to patients who meet the remaining clinical, pathological, biological, and treatment-related characteristics of these low-risk cohorts, with clinical and imaging assessment of the axilla substituting for pathological nodal confirmation. This represents an extrapolation beyond the populations prospectively evaluated in radiotherapy omission trials and requires prospective validation, just as the evidence from SLNB omission trials cannot be directly extrapolated to PBI.

This concept should not be interpreted as advocating PBI instead of radiotherapy omission, which remains an evidence-based option for appropriately selected patients. Rather, it questions whether omission of SLNB alone should automatically preclude PBI in patients who otherwise resemble these exceptionally low-risk populations.

Among patients undergoing omission of SLNB, those otherwise meeting contemporary radiotherapy omission criteria therefore represent the most appropriate population for prospective evaluation of PBI.

### 5.3. Can Patients at Risk of Occult Nodal Disease Be Better Identified?

A major concern regarding omission of SLNB before PBI is the possibility of unrecognized axillary metastases. This concern is justified, as approximately one in seven patients (14–15%) fulfilling SOUND or INSEMA eligibility criteria are found to harbor occult sentinel lymph node metastases when surgical staging is performed [10,11]. These patients, however, are biologically heterogeneous.

Numerous clinicopathological studies have confirmed that the probability of sentinel lymph node involvement is influenced by several readily identifiable factors, including tumor size, multifocality, lymphovascular invasion, tumor location, and the findings of preoperative axillary imaging [27,28]. A negative axillary ultrasound reduces, but does not eliminate, the probability of occult nodal involvement and therefore provides risk stratification rather than information equivalent to pathological staging [10,11,20]. More recently, prediction models integrating clinical and imaging characteristics have shown that combinations of these variables can identify subgroups with a very low probability of occult nodal disease [29,30]. These models integrate multiple clinicopathological and imaging variables rather than relying on a single predictor; however, their performance varies across populations, and none has been prospectively validated to select patients specifically for PBI following omission of SLNB.

These observations suggest that pathological nodal involvement should not be regarded as an unpredictable binary event but rather as a continuous risk that can increasingly be estimated before surgery. Refined prediction models integrating clinical characteristics, imaging findings, and established clinicopathological factors may allow more accurate estimation of an individual patient’s probability of occult nodal disease. Molecular subtypes and gene-expression profiles may provide complementary information regarding tumor biology and recurrence risk. However, genomic assays such as the 21-gene Oncotype DX Recurrence Score were developed primarily to estimate recurrence risk and chemotherapy benefit rather than occult axillary involvement and cannot currently substitute for pathological nodal staging.

The challenge is therefore not to identify all occult nodal metastases but to determine the level of residual risk at which pathological nodal staging continues to meaningfully influence patient selection for PBI.

### 5.4. Toward an Integrated Risk Assessment Framework

Patient selection for PBI has traditionally relied on predefined eligibility criteria. Increasingly, however, treatment decisions are based on individualized estimation of recurrence risk using complementary clinical, imaging, pathological, and molecular information [31]. Within this framework, pathological nodal staging becomes one component of overall risk assessment rather than an absolute prerequisite for treatment selection.

This paradigm shift is already evident in the field of radiotherapy omission. While early studies such as CALGB 9343 and PRIME II relied predominantly on conventional clinicopathological selection [23,24], more recent investigations including LUMINA and IDEA have incorporated biological and genomic information to identify patients with exceptionally low rates of local recurrence despite the omission of adjuvant radiotherapy [25,26]. Emerging genomic classifiers, including POLAR and ARTIC, further illustrate the potential of molecular profiling to estimate not only prognosis but also the individual benefit derived from radiotherapy itself [32,33]. Collectively, these developments indicate that local treatment decisions are likely to rely on integrated risk assessment rather than isolated pathological variables.

The same evolution may now be appropriate for patient selection for PBI. Rather than considering pathological nodal information an absolute prerequisite, selection strategies could integrate clinical characteristics, advanced multimodal imaging, established clinicopathological risk factors, molecular profiling, and individualized estimates of occult nodal involvement.

**Figure 1:**
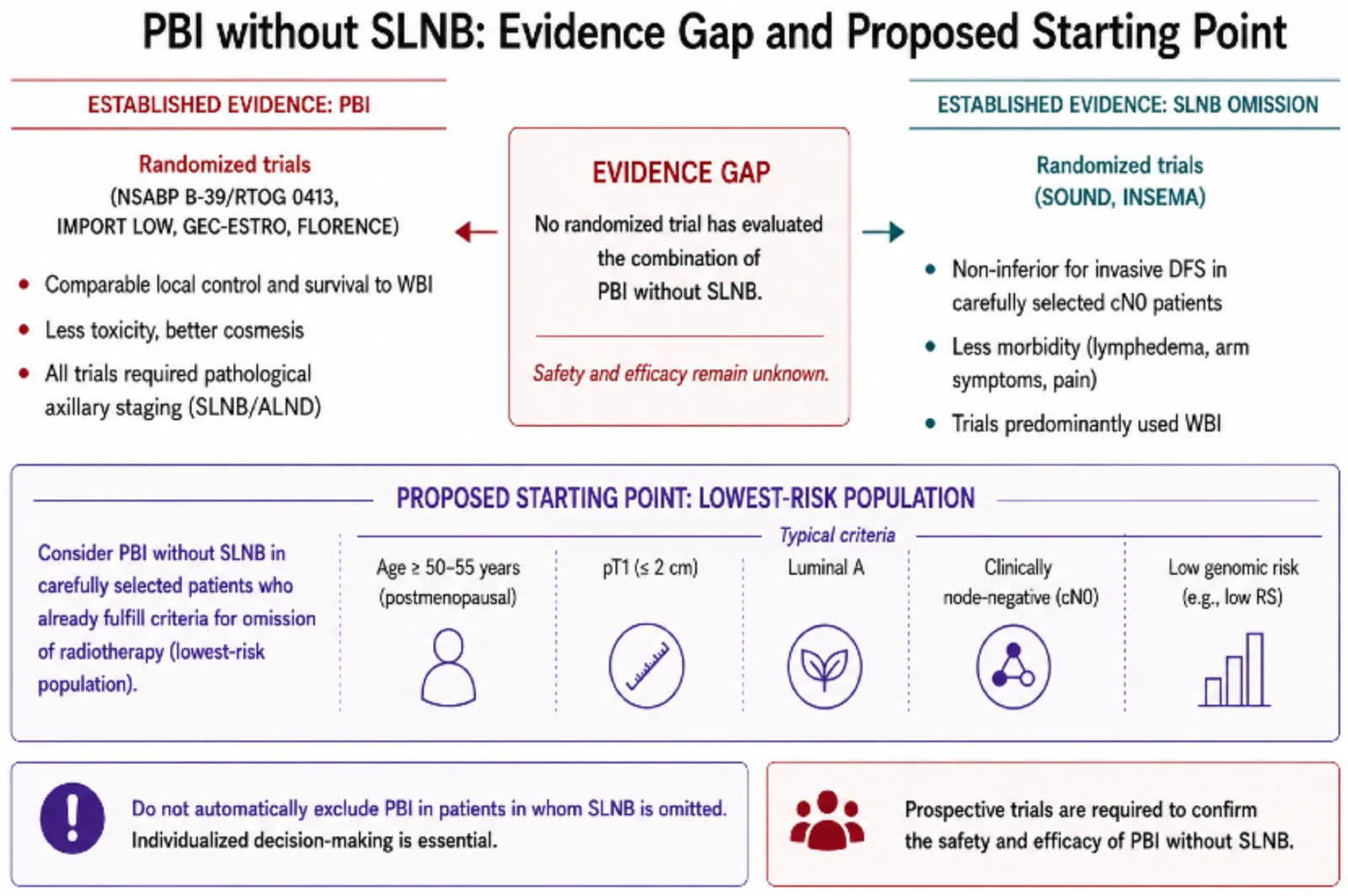
Evidence gap and proposed starting point for partial breast irradiation without sentinel lymph node biopsy. Abbreviations: PBI, partial breast irradiation; SLNB, sentinel lymph node biopsy; WBI, whole-breast irradiation; DFS, disease-free survival; cN0, clinically node-negative; RS, Recurrence Score.

From this perspective, omission of SLNB should not be viewed as creating an insurmountable barrier to PBI but rather as an opportunity to redefine patient selection using contemporary risk stratification. Ultimately, the objective should not be to replace one eligibility criterion with another but to identify patients in whom all available clinical, imaging, pathological, and biological information consistently indicates an exceptionally low probability of residual breast and axillary disease. This population is therefore the logical setting for prospective evaluation of PBI combined with omission of SLNB. The evidence gap and the proposed starting point for prospective evaluation of PBI without SLNB are summarized in Figure 1.

### 5.5. Future Directions

The convergence of evidence from randomized PBI trials, SLNB omission studies, and biologically guided radiotherapy de-escalation suggests that the traditional approach to patient selection requires re-evaluation. Available evidence remains insufficient to support routine implementation of PBI in patients undergoing omission of SLNB. Rather than immediate changes in clinical practice, these observations should guide the design of future prospective studies.

Such studies should focus on patients at the intersection of contemporary de-escalation strategies: those otherwise meeting stringent eligibility criteria for PBI, meeting established criteria for omission of SLNB, and exhibiting an exceptionally favorable clinical and biological risk profile. Incorporation of modern imaging, molecular profiling, and validated prediction models for occult nodal disease may further refine patient selection and reduce uncertainty regarding the absence of pathological nodal staging.

Prospective investigations should evaluate not only ipsilateral breast tumor recurrence but also regional control, patterns of failure, treatment-related toxicity, cosmetic outcomes, patient-reported quality of life, and health-economic endpoints. Particular attention should be paid to the potential influence of incidental axillary irradiation associated with whole-breast radiotherapy, as this represents one of the principal biological differences between existing SLNB omission trials and a strategy combining omission of SLNB with PBI.

Beyond regional dose coverage, radiation-field design may also have immunological consequences. Radiotherapy can both promote antitumor immune responses and impair immune organization, with the balance influenced by dose, fractionation, irradiated volume, and nodal exposure and potentially affecting circulating lymphocyte preservation, tumor-draining lymph node function, and tertiary lymphoid structures. Whether these mechanisms are clinically relevant to PBI following omission of SLNB remains unknown and should be regarded as hypothesis-generating rather than as evidence supporting this strategy [34].

The objective is not to demonstrate that pathological nodal staging is unnecessary but to determine in which patients it contributes meaningful additional information beyond current clinical, imaging, pathological, and molecular risk assessment. Further progress in local treatment de-escalation will depend on individualized risk prediction rather than any single eligibility criterion.

## 6. Conclusions

Partial breast irradiation (PBI) and omission of sentinel lymph node biopsy (SLNB) represent two of the most important advances in the de-escalation of locoregional treatment for early breast cancer. These strategies, however, have evolved independently under different clinical assumptions. The eligibility criteria for PBI were established in an era of routine pathological axillary staging, whereas randomized trials supporting omission of SLNB were conducted almost exclusively in patients receiving whole-breast irradiation. This has created an important evidence gap at the intersection of surgical and radiation treatment de-escalation, as the combination of omission of SLNB and PBI has never been prospectively evaluated [6,7,8,9,10,11,12,13].

The 2025 ASCO guideline recommends careful shared decision-making and preoperative multidisciplinary discussion when considering PBI following omission of SLNB, acknowledging the absence of direct randomized evidence for this combined approach [20]. Similarly, the updated 10-year analysis of IMPORT LOW and an editorial from the DEGRO Breast Cancer Expert Panel advise caution when considering PBI in the absence of pathological nodal staging [6,35]. The potential loss of incidental axillary irradiation from whole-breast tangential fields may be particularly relevant for tumors located in the lower quadrants [6].

The available evidence suggests that the role of pathological nodal information in PBI eligibility deserves reappraisal within the context of current breast cancer management. The increasing integration of high-quality imaging, biological characterization, molecular profiling, and individualized risk prediction offers an opportunity to move beyond reliance on isolated predefined eligibility criteria toward a more comprehensive assessment of local and regional recurrence risk. Omission of SLNB alone should not automatically preclude prospective evaluation of PBI in otherwise appropriately selected low-risk patients.

Among patients undergoing omission of SLNB, those otherwise meeting contemporary radiotherapy omission criteria provide the strongest biological and clinical rationale for prospective evaluation of PBI [23,24,25,26]. This refers to patients otherwise resembling these exceptionally low-risk populations, with clinical and imaging assessment of the axilla substituting for pathological nodal confirmation—an extrapolation that requires prospective validation. Radiotherapy omission itself remains an evidence-based option for appropriately selected patients [23,24,25,26]. Outside this lowest-risk population, combining PBI with omission of SLNB should remain a matter of careful multidisciplinary discussion and individualized decision-making until further evidence becomes available [20].

Ultimately, the objective is to develop robust patient selection strategies capable of identifying excellent candidates for PBI even in the absence of SLNB. Future prospective studies should determine how clinical characteristics, high-quality imaging, pathological features, molecular profiling, and individualized risk prediction can be integrated to accurately identify patients with an exceptionally low risk of residual breast and axillary disease. Such an approach would enable further refinement of personalized locoregional treatment while maintaining the excellent oncological outcomes established by modern de-escalation strategies.

## Figures and Tables

**Table 1:** Pivotal randomized trials of partial breast irradiation: patient selection, nodal staging, and outcomes.

Study	Design	N	Age	Tumor/Selection	Axillary Staging/Nodal Status	PBI Regimen	Median Follow-Up; Local Outcome
**IMPORT LOW** [6]	Phase III RCT, non-inferiority	2018	≥50 y	Unifocal invasive ductal carcinoma; ≤3 cm; margins ≥ 2 mm	Pathological staging; pN0–1 (≤3 positive nodes)	EBRT, 40 Gy/15 fx	10.0 y; 10-y IBTR 3.0% PBI vs. 2.8% WBI; non-inferior
**Florence** [7]	Phase III RCT	520	>40 y	Early-stage invasive BC ≤ 2.5 cm; predominantly low-risk	Surgical axillary staging; predominantly pN0	IMRT, 30 Gy/5 fx	10.7 y; 10-y IBTR 3.7% PBI vs. 2.5% WBI
**GEC-ESTRO** [8]	Phase III RCT, non-inferiority	1328	≥40 y	Stage 0–IIA; invasive BC/DCIS; ≤3 cm; negative margins	Pathological staging; pN0 or pN1mi	Multicatheter BT; HDR 30.1–32 Gy/7–8 fx; PDR 50 Gy	10.4 y; 10-y local recurrence 3.5% PBI vs. 1.6% WBI; non-inferior
**NSABP B-39/RTOG 0413** [9]	Phase III RCT, equivalence	4216	≥18 y	Stage 0–II; invasive BC/DCIS; ≤3 cm	Axillary staging for invasive BC; 0–3 positive nodes	BT or 3D-CRT, 34–38.5 Gy/10 fx	10.2 y; 10-y IBTR 4.6% PBI vs. 3.9% WBI; equivalence not demonstrated

**Abbreviations:** 3D-CRT, three-dimensional conformal radiotherapy; BC, breast cancer; BT, brachytherapy; DCIS, ductal carcinoma in situ; EBRT, external beam radiotherapy; fx, fractions; HDR, high dose rate; IBTR, ipsilateral breast tumor recurrence; IMRT, intensity-modulated radiotherapy; PBI, partial breast irradiation; PDR, pulsed dose rate; pN0, pathologically node-negative; pN1mi, nodal micrometastasis; RCT, randomized controlled trial; WBI, whole-breast irradiation.

**Table 2:** SLNB omission and radiotherapy omission studies relevant to combined locoregional de-escalation.

Study	Design	N	Age	Tumor/Biology	Nodal Assessment	Systemic Therapy	RT	Median Follow-Up; Key Outcome
**SOUND** [10]	RCT, non-inferiority	1463	≥18 y	Invasive BC ≤ 2 cm; predominantly HR+/HER2−	cN0 + negative axillary US; SLNB vs. no axillary surgery	According to standard practice	Mostly WBI (~80%); PBI minority	5.7 y; 5-y DDFS 98.0% no axillary surgery vs. 97.7% SLNB; non-inferior
**INSEMA** [11]	RCT, non-inferiority	5502	≥18 y	cT1–2 invasive BC; predominantly HR+/HER2−	cN0; SLNB vs. no axillary surgery	According to standard practice	WBI mandatory; PBI prohibited	6.1 y; 5-y iDFS 91.9% no axillary surgery vs. 91.7% SLNB; non-inferior
**CALGB 9343** [23]	RCT	636	≥70 y	T1, ER+	cN0; axillary dissection permitted but not required	Tamoxifen	WBI + boost vs. no RT	12.6 y; 10-y freedom from locoregional recurrence 90% no RT vs. 98% RT; no OS difference
**PRIME II** [24]	RCT	1326	≥65 y	T1–2 ≤ 3 cm; HR+; predominantly G1–2	pN0	Endocrine therapy	WBI vs. no RT	9.1 y; 10-y LR 9.5% no RT vs. 0.9% RT; no OS difference
**LUMINA** [25]	Prospective cohort	500	≥55 y	T1; G1–2; luminal A: ER+, PR > 20%, HER2−, Ki-67 ≤ 13.25%	pN0	Endocrine therapy	No RT	5 y; 5-y LR 2.3% without RT
**IDEA** [26]	Prospective cohort	200	50–69 y, postmenopausal	pT1; ER+/PR+/HER2−; RS ≤ 18	pN0	Endocrine therapy	No RT	5.2 y; 5-y freedom from any recurrence 99% without RT

Abbreviations: BC, breast cancer; cN0, clinically node-negative; DDFS, distant disease-free survival; ER, estrogen receptor; G, grade; HER2, human epidermal growth factor receptor 2; HR, hormone receptor; iDFS, invasive disease-free survival; LR, local recurrence; OS, overall survival; PBI, partial breast irradiation; pN0, pathologically node-negative; RCT, randomized controlled trial; RS, recurrence score; RT, radiotherapy; SLNB, sentinel lymph node biopsy; US, ultrasound; WBI, whole-breast irradiation.

## Data Availability

No new data were created or analyzed in this study. Data sharing is not applicable to this article.

## Abbreviations

ASCO	American Society of Clinical Oncology
GEC-ESTRO	Groupe Européen de Curiethérapie–European Society for Radiotherapy and Oncology
INSEMA	Intergroup Sentinel Mamma Trial
PBI	Partial breast irradiation
SLNB	Sentinel lymph node biopsy
SOUND	Sentinel node vs. Observation after axillary UltraSouND
WBI	Whole-breast irradiation
